# Supplementary material for: Genetic risk score for coronary artery calcification and its predictive ability for coronary artery disease
Source: Am J Prev Cardiol. 2024 Oct 10;20:100884. doi: 10.1016/j.ajpc.2024.100884 (PMC11525111; doi:10.1016/j.ajpc.2024.100884)
Supplement: Supplementary file 1 [file mmc1.pdf]

Supplementary materials

Genetic risk score for coronary artery calcification and its predictive ability for coronary artery disease

Pashupati P. Mishra*[1,2,3], Binisha H. Mishra[1,2,3], Leo-Pekka Lyytikäinen[1,2,3], Sirkka Goebeler[4], Mika Martiskainen[4,5], Emma Hakamaa[1,2], Marcus E. Kleber[6,7], Graciela E. Delgado[6], Winfried März[6,8], Mika Kähönen[2,9], Pekka J. Karhunen[1,2,3], Terho Lehtimäki[1,2,3]

Affiliations:

[1]Department of Clinical Chemistry, Faculty of Medicine and Health Technology, Tampere University, Tampere, Finland

[2]Finnish Cardiovascular Research Center Tampere, Faculty of Medicine and Health Technology, Tampere University, Tampere, Finland

[3]Department of Clinical Chemistry, Fimlab Laboratories, Tampere, Finland

[4]Forensic Medicine, Finnish Institute for Health and Welfare, Helsinki, Finland

[5]Faculty of Medicine and Health Technology, Tampere University and Fimlab Laboratories, Tampere, Finland

[6]Vth Department of Medicine, University Medicine Mannheim, Medical Faculty Mannheim of the University of Heidelberg, Mannheim, Germany

[7]SYNLAB MVZ Humangenetik Mannheim, Mannheim, Germany

[8]Synlab Academy, SYNLAB Holding Deutschland GmbH, Mannheim and Augsburg, Germany

[9]Department of Clinical Physiology, Tampere University Hospital, Tampere Finland.

*Correspondence to:

Pashupati P. Mishra, PhD

Email: pashupati.mishra@tuni.fi

1. Genetic risk loci for coronary artery calcification (CAC)

The independent lead SNPs from 11 different loci associated with coronary artery calcification, reported by the recent genome-wide association study (GWAS) by [1] is presented in Table S1. Three of the 11 SNPs (rs9349379, rs9515203 and rs7412) were also among the 1,745,179 SNPs used to construct metaGRS, a genetic risk score for CAD [2].

Table S1: List of the 11 independent SNPs associated with coronary artery calcification with their imputation quality score (R^2) and minor allele frequency (MAF) in the three studied cohorts. The SNPs marked with asterisk were also among the 1,745,179 SNPs used to construct metaGRS.

CHR	*POSITION*	*ID*	*rsid*	*REF*	*ALT*	*LURIC*		*TSDS*		*TVS*	
						R^2	*MAF*	R^2	*MAF*	R^2	*MAF*
6	12903957	6:12903957	rs9349379*	A	G	1.00	0.42	1.00	0.46	1.00	0.47
6	132095002	6:132095002	rs3844006	C	T	0.99	0.23	1.00	0.32	1.00	0.33
7	45960645	7:45960645	rs2854746	G	C	0.96	0.41	0.95	0.35	0.97	0.34
9	22124744	9:22124744	rs4977575	C	G	0.98	0.47	1.00	0.45	0.98	0.48
10	44515716	10:44515716	rs10899970	G	A	1.00	0.48	1.00	0.48	1.00	0.49
10	63836088	10:63836088	rs9633535	C	T	1.00	0.40	1.00	0.36	1.00	0.32
10	75917431	10:75917431	rs10762577	A	G	0.99	0.23	0.98	0.29	0.97	0.30
12	4486618	12:4486618	rs11063120	A	G	0.93	0.15	0.79	0.25	0.87	0.22
13	111049623	13:111049623	rs9515203*	T	C	1.00	0.24	0.87	0.28	0.93	0.27
15	79123946	15:79123946	rs7182103	G	T	1.00	0.42	1.00	0.31	0.99	0.34
19	45412079	19:45412079	rs7412*	C	T	1.00	0.07	1.00	0.05	0.92	0.07

Abbreviations: LURIC, the Ludwigshafen Risk and Cardiovascular Health Study; TSDS, the Tampere Sudden Death Study; TVS, the Tampere Vascular Study.

Table S2: The independent lead SNPs from 11 different loci associated with coronary artery calcification with their imputation quality score (R^2) and minor allele frequency (MAF) in the three studied cohorts for CAD cases and controls separately. The SNPs marked with asterisk were also among the 1,745,179 SNPs used to construct metaGRS.

CHR	*POSITION*	*ID*	*rsid*	*REF*	*ALT*	*LURIC*			*TSDS*			*TVS*		
						R^2	*MAF*		*R^2*	*MAF*		*R^2*	*MAF*	
							CAD cases	*Controls*		*CAD cases*	*Controls*		*CAD cases*	*Controls*
6	12903957	6:12903957	rs9349379*	A	G	1.00	0.43	0.39	1.00	0.49	0.43	1.00	0.46	0.50
6	132095002	6:132095002	rs3844006	C	T	0.99	0.23	0.24	1.00	0.31	0.34	1.00	0.34	0.27
7	45960645	7:45960645	rs2854746	G	C	0.96	0.43	0.40	0.95	0.37	0.34	0.97	0.31	0.40
9	22124744	9:22124744	rs4977575	C	G	0.98	0.45	0.48	1.00	0.49	0.40	0.98	0.47	0.33
10	44515716	10:44515716	rs10899970	G	A	1.00	0.47	0.50	1.00	0.48	0.48	1.00	0.49	0.49
10	63836088	10:63836088	rs9633535	C	T	1.00	0.40	0.39	1.00	0.34	0.39	1.00	0.35	0.23
10	75917431	10:75917431	rs10762577	A	G	0.99	0.22	0.26	0.98	0.29	0.31	0.97	0.29	0.34
12	4486618	12:4486618	rs11063120	A	G	0.93	0.15	0.14	0.79	0.26	0.26	0.87	0.24	0.24
13	111049623	13:111049623	rs9515203*	T	C	1.00	0.23	0.27	0.87	0.26	0.29	0.93	0.23	0.32
15	79123946	15:79123946	rs7182103	G	T	1.00	0.42	0.45	1.00	0.30	0.34	0.99	0.33	0.35
19	45412079	19:45412079	rs7412*	C	T	1.00	0.07	0.08	1.00	0.05	0.05	0.92	0.06	0.06

2. Calculation of metaGRS for LURIC, TSDS and TVS participants.

The metaGRSs in all the three cohorts (LURIC, TVS and TSDS) were calculated using the GWAS summary statistics of the 1,745,179 SNPs associated with CAD [2]. The summary statistics of the 1,745,179 SNPs was downloaded from the polygenic score (PGS) catalog [https://www.pgscatalog.org/score/PGS000018/]. In LURIC (n=2742), after excluding SNPs with MAF < 1% and $R^2 < 0.3$, we ended up with a total of 1,581,873 SNPs available for calculation of metaGRS. In TSDS (n=660), after excluding SNPs with MAF < 1% and $R^2 < 0.3$, we ended up with a total of 1,486,341 SNPs available for calculation of metaGRS. Similarly, in TVS (n=133), after excluding SNPs with MAF < 2% and $R^2 < 0.3$, we ended up with a total of 1,287,176 SNPs available for calculation of metaGRS. Even though all the variants used to construct original metaGRS had MAF > 0.1%, we used different MAF thresholds depending on the sample size of the studied cohorts. The metaGRSs were then calculated as sum of effect allele dosages or counts of the SNPs weighted by their corresponding effect sizes.

3. Genetic risk scores for coronary artery calcification (CAC GRS) and their distribution among CAD cases and controls across the three studied cohorts.

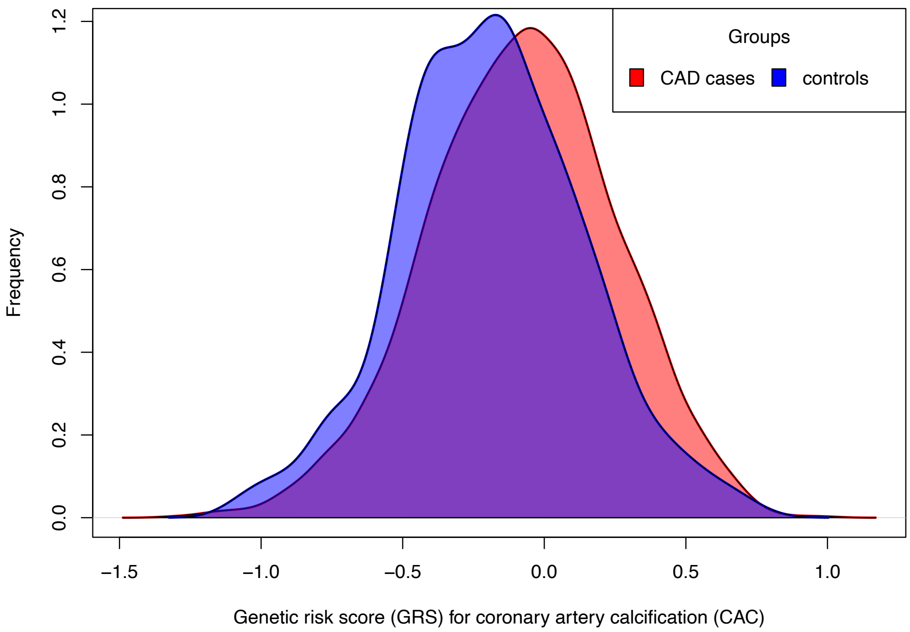

Figure S1: Density curves of standardized genetic risk scores for coronary artery calcification (CAC GRS) in the Ludwigshafen Risk and Cardiovascular Health (LURIC) study participants stratified by case-control status of coronary artery disease (CAD).

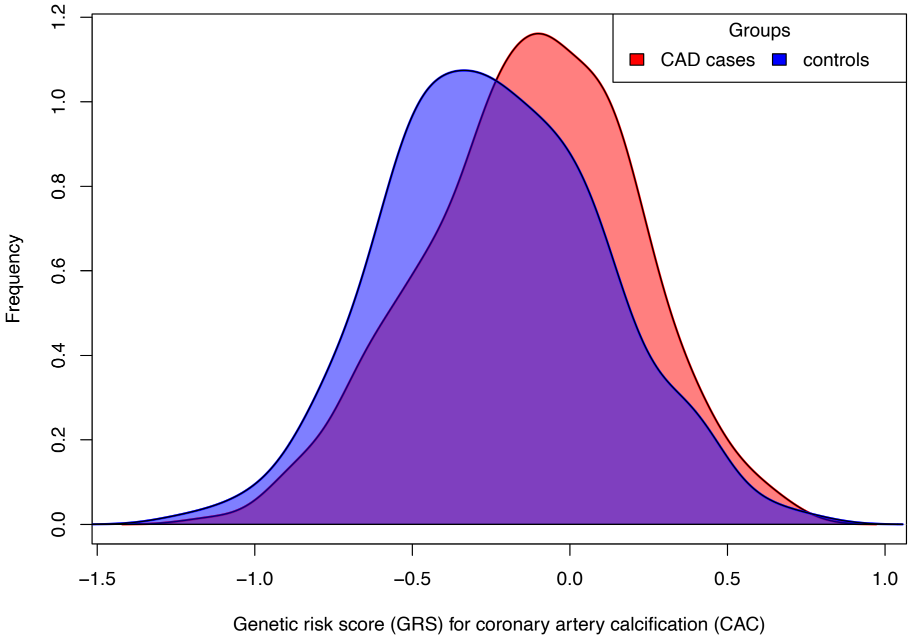

Figure S2: Density curves of standardized genetic risk scores for coronary artery calcification (CAC GRS) in the Tampere Sudden Death Study (TSDS) participants stratified by case-control status of coronary artery disease (CAD).

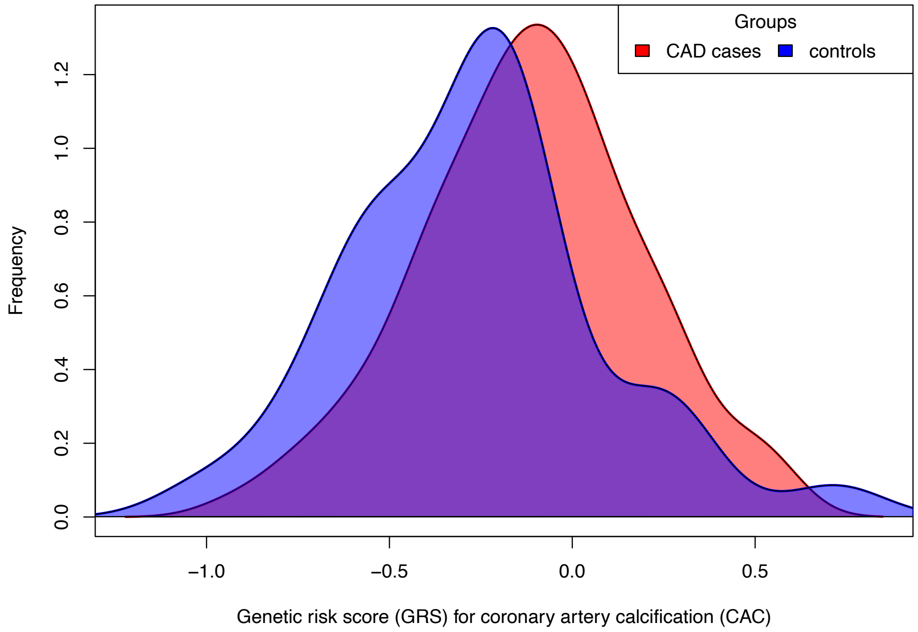

Figure S3: Density curves of standardized genetic risk scores for coronary artery calcification (CAC GRS) in the Tampere Vascular Study (TVS) participants stratified by case-control status of coronary artery disease (CAD).

4. Sex- and age-stratified association analysis of the genetic risk score for coronary artery calcification (CAC GRS) and clinical cardiovascular disease (CVD) risk factors with coronary artery disease (CAD).

Table S3. Male specific Association of the genetic risk score for coronary artery calcification (CAC GRS) and clinical cardiovascular disease (CVD) risk factors with coronary artery disease (CAD) in the Ludwigshafen Risk and Cardiovascular Health (LURIC) study participants.

	**Model 1**		**Model 2**		**Model 3**	
**Risk factors**	**OR (95% CI)**	**P-value**	**OR (95% CI)**	**P-value**	**OR (95% CI)**	**P-value**
Age	1.05 (1.04–1.06)	$< 2 \times 10^{-16}$	1.05 (1.04 –1.07)	$< 2 \times 10^{-16}$	1.06 (1.05–1.08)	$< 2 \times 10^{-16}$
Body mass index (BMI)	0.98 (0.95–1.01)	0.12	-	-	-	-
Systolic blood pressure	-	-	1.00 (1.00 – 1.01)	0.45	1.00 (1.00–1.01)	0.32
Total cholesterol	-	-	1.00 (0.99 – 1.00)	0.89	1.00 (1.00–1.003)	0.80
HDL cholesterol	-	-	0.97 (0.96 – 0.99)	1.8×10^{-05}	0.97 (0.96–0.99)	8.3×10^{-05}
Smoking habit (yes)	-	-	1.76 (1.35 – 2.30)	3.2×10^{-05}	1.70 (1.28–2.25)	0.0002
Hypertension medication (yes)	-	-	1.32 (1.02 – 1.71)	0.04	1.20 (0.91–1.57)	0.19
CAC GRS	1.49 (1.33–1.66)	2.7×10^{-12}	1.47 (1.30 – 1.66)	6.9×10^{-10}	1.23 (1.08–1.40)	0.002
metaGRS	-	-	-	-	2.06 (1.80–2.37)	$< 2 \times 10^{-16}$

Table S4. Female specific Association of the genetic risk score for coronary artery calcification (CAC GRS) and clinical cardiovascular disease (CVD) risk factors with coronary artery disease (CAD) in the Ludwigshafen Risk and Cardiovascular Health (LURIC) study participants.

	Model 1		Model 2		Model 3	
**Risk factors**	**OR (95% CI)**	**P-value**	**OR (95% CI)**	**P-value**	**OR (95% CI)**	**P-value**
Age	1.04 (1.02–1.05)	2.2 x 10^{-06}	1.04 (1.02 –1.06)	1.6 x 10^{-05}	1.05 (1.03–1.07)	1.7 x 10^{-07}
Body mass index (BMI)	1.00 (0.97–1.03)	0.99	-	-	-	-
Systolic blood pressure	-	-	1.00 (1.00 – 1.01)	0.21	1.00 (1.00–1.01)	0.24
Total cholesterol	-	-	1.00 (1.00 – 1.01)	0.10	1.00 (1.00–1.01)	0.36
HDL cholesterol	-	-	0.98 (0.97 – 0.99)	0.007	0.98 (0.97–1.00)	0.01
Smoking habit (yes)	-	-	1.98 (1.39 – 2.84)	0.0002	1.83 (1.27–2.67)	0.001
Hypertension medication (yes)	-	-	1.36 (0.95 – 1.94)	0.09	1.28 (0.88–1.86)	0.19
CAC GRS	1.39 (1.20–1.61)	1.0 x 10^{-05}	1.31 (1.12 – 1.54)	0.0007	1.08 (0.91–1.28)	0.36
metaGRS	-	-	-	-	2.21 (1.82–2.70)	5.2 x 10^{-15}

Table S5. Association of the genetic risk score for coronary artery calcification (CAC GRS) and clinical cardiovascular disease (CVD) risk factors with coronary artery disease (CAD) in the Ludwigshafen Risk and Cardiovascular Health (LURIC) study participants aged 40 years and above.

	Model 1		Model 2		Model 3	
**Risk factors**	**OR (95% CI)**	**P-value**	**OR (95% CI)**	**P-value**	**OR (95% CI)**	**P-value**
Age	1.04 (1.03–1.05)	7.8 x 10^{-16}	1.05 (1.03 –1.06)	2.0 x 10^{-15}	1.06 (1.04–1.07)	< 2 x 10^{-16}
Sex (male)	3.57 (2.96–4.32)	< 2 x 10^{-16}	2.80 (2.24 – 3.52)	< 2 x 10^{-16}	3.22 (2.54–4.09)	< 2 x 10^{-16}
Body mass index (BMI)	0.98 (0.96–1.01)	0.12	-	-	-	-
Systolic blood pressure	-	-	1.00 (1.00 – 1.01)	0.13	1.00 (1.00–1.01)	0.09
Total cholesterol	-	-	1.00 (1.00 – 1.004)	0.26	1.00 (0.99–1.00)	0.39
HDL cholesterol	-	-	0.98 (0.97 – 0.99)	2.0 x 10^{-06}	0.98 (0.97–0.99)	1.1 x 10^{-05}
Smoking habit (yes)	-	-	1.77 (1.43 – 2.19)	1.4 x 10^{-07}	1.70 (1.36–2.12)	3.1 x 10^{-06}
Hypertension medication (yes)	-	-	1.32 (1.07 – 1.63)	0.01	1.23 (0.98–1.53)	0.07
CAC GRS	1.43 (1.31–1.57)	5.5 x 10^{-15}	1.39 (1.26 – 1.53)	3.9 x 10^{-11}	1.16 (1.05–1.28)	0.005
metaGRS	-	-	-	-	2.10 (1.87–2.34)	< 2 x 10^{-16}

References:

1. Kavousi, M., Bos, M. M., Barnes, H. J., Lino Cardenas, C. L., Wong, D., Lu, H., … Miller, C. L. (2023). Multi-ancestry genome-wide study identifies effector genes and druggable pathways for coronary artery calcification. *Nature Genetics*, *55*(10), 1651–1664. https://doi.org/10.1038/s41588-023-01518-4
2. Inouye, M., Abraham, G., Nelson, C. P., Wood, A. M., Sweeting, M. J., Dudbridge, F., … Samani, N. J. (2018). Genomic Risk Prediction of Coronary Artery Disease in 480,000 Adults: Implications for Primary Prevention. *Journal of the American College of Cardiology*, *72*(16), 1883–1893. https://doi.org/10.1016/j.jacc.2018.07.079
